# Prosocial Orientations: Distinguishing Compassionate Goals From Other Constructs

**DOI:** 10.3389/fpsyg.2020.538165

**Published:** 2020-09-17

**Authors:** Amy Canevello, Jennifer Crocker

**Affiliations:** ^1^Department of Psychological Science, University of North Carolina at Charlotte, Charlotte, NC, United States; ^2^Department of Psychology, The Ohio State University, Columbus, OH, United States

**Keywords:** prosocial orientations, compassionate goals, interpersonal, giving, gratitude

## Abstract

The compassionate goals scale was developed to assess the intentions underlying prosocial behaviors. Over the past 10 years, it has been shown to predict prosociality. However, research has not yet examined how compassionate goals relate to other measures of prosocial orientations or demonstrated that compassionate goals predict unique variance beyond them. Three studies addressed this shortcoming in the existing literature. Across studies, participants completed measures of compassionate goals, compassionate love, communal orientation, communion, unmitigated communion, and empathic concern. The participants also reported giving to strangers (study 1) and giving to close others (study 2). Study 3 was dyadic in nature—the participants reported their reasons for giving to friends and gratitude, and friends reported their gratitude toward the participants. Despite strong correlations between the compassionate goals scale and other prosocial orientation measures, compassionate goals items are empirically distinct from items assessing other prosocial orientations. The compassionate goals measure accounts for unique variance in giving, reasons for giving, and gratitude. Path analyses support a dyadic process—that compassionate goals predict more other-focused reasons for giving, which then predict friends’ gratitude toward the participants. While the compassionate goals measure does overlap with other well-established and commonly used measures of prosocial orientation measures, it accounts for unique variance in giving-related outcomes, suggesting that intentions are an important aspect of prosocial orientations.

## Introduction

Although people often act out of self-interest, they also have the capacity to engage in prosocial behaviors, acting in ways that benefit others [see Crocker et al. (2017a), for a review]. A long history of research on helping behavior, altruism, and other forms of prosocial behavior focuses on who behaves prosocially and under what circumstances (Darley and Batson, 1973; Penner et al., 2005). Prosocial behavior has received renewed attention as researchers aim to better understand not only individual differences and moderators of prosocial behavior but also the mechanisms that account for its effects on health and well-being (e.g., Mikulincer and Shaver, 2010; Crocker et al., 2017a; Seppälä et al., 2017).

Despite receiving considerable empirical attention, the motivations that underlie prosocial behavior are debated (e.g., Batson, 2017). Much research and theory assume that people are primarily self-interested and that they engage in prosocial behavior to benefit the self (e.g., Cialdini et al., 1987). Indeed people who act prosocially benefit through reciprocity, enhanced reputation, reduced distress, and inclusion in groups and relationships (e.g., Leary, 1995; Leary and Baumeister, 2000).

While it is undoubtedly true that prosocial behaviors are sometimes energized by self-interest, research shows that people sometimes engage in prosocial behaviors because they genuinely care about others (Crocker et al., 2017a). The egosystem–ecosystem theory proposed that all humans have the capacity for two types of social motivation (e.g., Crocker and Canevello, 2015, 2017). When motivated by the egosystem, people focus on satisfying their own needs and desires and, if they think about others’ needs at all, view others’ needs and desires as secondary or in competition with their own. In this view, other people are either means or obstacles for satisfaction of needs and desires or they are irrelevant. More important for the present investigation is the fact that, when motivated by the ecosystem, people focus on the well-being of others in addition to themselves and view others not as means or obstacles but as human beings with needs and desires that are important to them. They assume that people are interconnected in an interpersonal ecosystem, so the satisfaction of others’ needs benefits the interpersonal ecosystem and often, indirectly, the self. They care about and want to support the well-being of others in their interpersonal ecosystem.

Ecosystem motivation suggests a paradigm for giving that is distinct from those offered by other conceptions of altruistic motivations for helping. Traditionally, altruism had been framed from a zero-sum perspective and is typically assumed to involve giving aid to others that is costly to the self (e.g., Van Lange et al., 1997; Feeney and Collins, 2003; Impett et al., 2005; Batson, 2010). In contrast, giving in the ecosystem is nonzero-sum, driven by “otherish motivation” or wanting to benefit others because one cares about their well-being (Crocker et al., 2017a). Research supports this assertion: Compassionate goals are explicitly related to nonzero-sum views of relationships, in which people search for solutions to problems that are good for others and for the self (Crocker et al., 2017b).

Crocker and Canevello (2008) developed a measure of compassionate goals to assess ecosystem motivation as revealed by intentions to be supportive and constructive and not harm others. A decade of research has demonstrated that this measure is reliable, has convergent and divergent validity with other constructs such as self-compassion, has secure attachment, has spiritual transcendence, has self-consciousness (Crocker and Canevello, 2008), and predicts people’s own and others’ relational outcomes and personal well-being (Canevello and Crocker, 2010, 2011; Crocker et al., 2010). Compassionate goals are associated with more prosocial behavior, both cross-sectionally and longitudinally. When people are higher in compassionate goals, they view relationships as working in nonzero-sum ways, have a more cooperative mindset, and feel at ease and connected to others (Canevello and Crocker, 2017), and they give more support to others and are more responsive to them (e.g., Canevello and Crocker, 2010; Crocker et al., 2010). These prosocial behaviors, in turn, have consequences for their own and others’ experiences: When people give, others reciprocate, leading to better relationships and personal well-being.

Despite evidence of the reliability and the validity of the compassionate goals scale (e.g., Crocker and Canevello, 2012, 2015, 2017), questions remain about its contribution to research on prosocial motivation. Specifically, little is known about the extent to which the compassionate goals measure overlaps with other measures of prosocial orientations or whether its focus on intentions captures something unique about prosocial motivation that have not been isolated by existing measures. The compassionate goals scale shares similarities with a number of existing measures including compassionate love (Fehr and Sprecher, 2008), communal orientation (Clark et al., 1987), communion (Spence et al., 1979), unmitigated communion (Fritz and Helgeson, 1998), and empathic concern (Davis, 1983). These measures all predict prosocial behaviors but do not explicitly focus solely on the motivations or the intentions that energize prosocial behavior. Psychologists from Lewin (1951) onward have argued for the critical role of motives in explaining behavior. Understanding the motivation and the intentions underlying behavior provides insight into the nature, frequency, effectiveness of behavior, and conditions under which it occurs (Batson, 2010). If these other measures in part assess the same underlying orientations, they should be highly correlated with the compassionate goals scale, and these various measures may be interchangeable with it. If, however, the intentions assessed by the compassionate goals scale represent something unique about prosocial orientation, then the items assessing compassionate goals should be empirically distinguishable from the items on existing scales. Moreover, the compassionate goals scale should account for variance in indicators of prosocial behavior, such as giving, beyond that accounted for by existing measures.

Because theory suggests that compassionate goals are energized by the desire to promote others’ well-being (Crocker and Canevello, 2008), the compassionate goals scale should predict people’s reasons for giving to others: people higher in compassionate goals should be more likely to give to others in order to benefit others’ well-being. Giving, for this reason, should create relationships characterized by gratitude. Recipients should feel more grateful because when others give with the intention to support the recipients’ well-being, the support given should match the recipients’ needs or desires (e.g., Sibicky et al., 1995; Batson, 2017). Because compassionate goals lead to better relationships characterized by mutual support and responsiveness, people with compassionate goals may also feel more gratitude.

In this investigation, we compare the compassionate goals scale with other measures that have well-established empirical links to prosocial behaviors and may appear to assess the same construct—measures of compassionate love, communal orientation, communion, unmitigated communion, and empathic concern (Batson, 2009, 2017; Gebauer et al., 2013; Collins et al., 2014; Fehr et al., 2014; Le et al., 2018). These measures assess distinct components of prosocial orientation. The compassionate love scale was intended to capture an enduring prosocial and other-oriented type of love characterized by sympathy, sacrifice, and providing comfort to strangers in need (Fehr and Sprecher, 2008). The communal orientation scale reflects a sense of responsibility for another’s welfare, either out of concern, feelings of obligation, or wanting to please the other, with the expectation that others will reciprocate (Clark et al., 1987). The communion measure assesses an orientation toward establishing and maintaining connections with others and is characterized by a focus on others, being attuned to others’ feelings, and having empathy for and helping them (Bakan, 1966; Spence et al., 1979; Fritz and Helgeson, 1998). The unmitigated communion measure assesses an excessive concern with others, wherein others’ needs and well-being are prioritized over one’s own needs and well-being (Fritz and Helgeson, 1998; Helgeson and Fritz, 1998). Items assessing empathic concern focus on emotional responses to observing another’s distress that reflect a sense of warmth and concern for others (Davis, 1983). While all of these measures may incorporate prosocial intentions to some extent, none of them focuses solely on intentions toward others. The compassionate goals scale was developed to measure the intentions underlying prosocial behaviors, which are not clearly the focus of other operationalizations of prosocial orientations.

The present studies had four primary aims. First, we assess the degree of empirical overlap between compassionate goals and existing measures of prosocial orientations (studies 1–3). Second, we test whether the items on the compassionate goals scale are empirically separable from the items on each of the other measures with confirmatory factor analyses (CFAs; studies 1 and 2). Third, we test whether compassionate goals predict indicators of prosocial behavior including prosocial giving, reasons for giving, and recipients’ responses to giving (studies 1–3). Fourth, we test whether compassionate goals indirectly predict others’ experience of gratitude through the reasons people have for giving to relationship partners (study 3). Thus, the dyadic nature of study 3 goes beyond self-reported prosocial behavior to capture others’ responses to prosocial behavior. Furthermore, we examine prosocial giving across relationships—in strangers (study 1), close others (study 2), and friends (study 3). Informed consent was obtained from the participants in all three studies; data and syntax can be found at https://osf.io/gxyf7/?view_only=3214297b709344c380b92577ae17167e.

## Study 1

Study 1 had three aims. First, we examined the degree of empirical overlap among compassionate goals and compassionate love, communal orientation, communion, unmitigated communion, and empathic concern by computing correlations between them. Second, we tested the distinctiveness of the compassionate goals scale items from items on each of the other measures by conducting CFAs. Third, we examined whether compassionate goals explain the variance in a range of self-reported altruistic behaviors toward strangers and whether that variance is distinct from the variance explained by each of the other scales. We examined the ability of these measures to predict responses to a widely used scale assessing altruism as a wide range of behaviors. Because compassionate goals have been positively correlated with social desirability and gender in previous studies (e.g., Crocker and Canevello, 2008; Canevello and Crocker, 2010), we also tested whether links between compassionate goals and altruistic giving to strangers could be accounted for by socially desirable responding and gender.

### Method

#### Participants

Introductory Psychology students (*N* = 571) at a large Midwestern university completed questionnaires in a laboratory session. Fifty-five percent were female; the participants’ ages ranged from 18 to 23 (*M* = 18.77 years, *SD* = 1.18). The sample was 75% White/Caucasian. Data were collected in a single academic term; we collected as much data as possible during that time. This sample provided adequate power for confirmatory factor analyses (Tabachnick and Fidell, 2013), and *post hoc* power analyses using G^∗^Power (Faul et al., 2007) suggest that this sample provided power of 0.84 to detect small effects (*r* = 0.11) in the correlation and the regression analyses described below.

#### Measures

The participants completed measures of compassionate goals, compassionate love, communal orientation, communion, unmitigated communion, and empathic concern. They also completed measures of altruistic giving to strangers, social desirability, and demographics. Additional measures not germane to the present investigation were also included. The internal reliabilities (αs) for all study 1 scales are reported in Table 1.

**TABLE 1:** Correlations, means, standard deviations, and internal reliabilities for Study 1 variables.

	1	2	3	4	5	6	7	8	*M*	*SD*
(1) Compassionate goals	*0.84*								3.91	0.55
(2) Compassionate love	0.47	*0.95*							3.30	0.74
(3) Communal orientation	0.52	0.56	*0.78*						3.72	0.49
(4) Communion	0.46	0.57	0.56	*0.77*					3.34	0.57
(5) Unmitigated communion	0.53	0.51	0.65	0.49	*0.83*				3.92	0.56
(6) Empathic concern	0.49	0.65	0.65	0.51	0.61	*0.79*			3.74	0.68
(7) Altruistic giving to strangers	0.33	0.48	0.30	0.33	0.27	0.31	*0.87*		3.00	0.64
(8) Social desirability	0.28	0.32	0.20	0.19	0.28	0.21	0.21	*0.73*	0.45	0.15
(9) Gender	0.23	0.26	0.26	0.17	0.21	0.34	0.08	0.09		

*N = 571. Internal reliabilities are reported on the diagonal in italics. Gender was correlated with altruistic giving to strangers (p = 0.050) and social desirability (p = 0.032). All other correlations were also statistically significant (p < 0.001). Social desirability scores ranged from 0 to 1, with higher scores indicating higher social desirability. Gender was coded such that 1 = male and 2 = female. For all other measures, items were rated on scales from 1 to 5, with higher scores indicating higher levels of the construct.*

Compassionate goals were assessed using a modified measure from Crocker and Canevello (2008). Items began with the phrase “In general, how much do you want or try to” and were rated on a scale ranging from 1 (not at all) to 5 (extremely). Eleven items assessed compassionate goals. The sample items include “Be supportive of others” and “Avoid doing anything that would be harmful to others.”

Compassionate love for humanity was assessed using the measure from Fehr and Sprecher (2008). The participants rated 21 items on a scale from 1 (not at all true of me) to 5 (very true of me). The sample items include “I tend to feel compassion for people, even though I do not know them” and “I feel considerable compassionate love for people from everywhere.”

Communal orientation was measured using the scale developed by Clark et al. (1987). The participants rated 14 items in terms of how characteristic each item was of them on a scale ranging from 1 (extremely uncharacteristic) to 5 (extremely characteristic). The sample items include “When making a decision, I take other people’s needs and feelings into account” and “I expect people I know to be responsive to my needs and feelings.”

Communion was measured using the subscale from the extended version of the Personal Attributes Questionnaire (Spence et al., 1979). The participants rated themselves on eight attributes using a 1-to-5 scale. The sample items include “Not at all emotional/very emotional” and “Not at all helpful to others/very helpful to others.”

Unmitigated communion was measured using the scale developed by Fritz and Helgeson (1998). The participants rated nine items on a scale ranging from 1 (strongly disagree) to 5 (strongly agree). The sample items include “I always place the needs of others above my own” and “I cannot say no when someone asks for help.”

Empathic concern was measured using the empathic concern subscale of the Interpersonal Reactivity Index (Davis, 1983). Seven items were rated on a scale from 1 (does not describe me well) to 5 (describes me very well). The sample items include “I am often quite touched by things that I see happen” and “I often have tender, concerned feelings for people less fortunate than me.”

Altruistic giving to strangers was measured using the scale from Rushton et al. (1981), in which the participants rate how much they have committed 21 acts of giving to strangers on a scale from 1 (never) to 5 (very often). The sample items include “I have given money to a charity” and “I have helped carry a stranger’s belongings.”

Social desirability was measured with the Marlowe–Crowne Social Desirability Scale (Crowne and Marlowe, 1964). The participants were asked whether or not they endorse each of the 33 statements. The sample items include “Before voting, I thoroughly investigate the qualifications of the candidate” and “No matter who I am talking to, I am always a good listener.”

### Results

#### Overview of Analyses

We conducted study 1 analyses in three phases. In phase 1, we examined the zero-order correlations among compassionate goals and the other measures of prosocial orientations. In phase 2, we conducted five pairs of CFAs, each comparing the fit of (1) a model in which items from the compassionate goals scale loaded onto the same factor as items from the other measure with (2) a model in which items from the two scales loaded onto separate factors. In phase 3, we examined whether compassionate goals explain variance in altruistic giving to strangers and whether that variance is distinct from that explained by each of the other scales. We did this by first examining whether compassionate goals and other measures of prosocial orientations were correlated with altruistic giving to strangers. Next, we tested whether compassionate goals explain unique variance in altruistic giving to strangers when entered with each of the other prosocial orientations individually. Finally, we tested whether compassionate goals captured unique variance in altruistic giving to strangers when all of the prosocial orientations measures were included in a single analysis and examined whether these findings were due to social desirability and gender.

#### Associations Between Compassionate Goals and Other Prosocial Orientation Measures

First, we examined correlations among compassionate goals and measures of compassionate love, communal orientation, communion, unmitigated communion, and empathic concern. As shown in the first column of Table 1, compassionate goals were correlated with each of the other measures of prosocial orientations, 0.46 ≤ *r* ≤ 0.53. This overlap is unsurprising, given that each assesses some aspect of prosocial orientations.

#### Are Items Assessing Compassionate Goals Scale Empirically Separable From Items Assessing Other Prosocial Orientations?

In phase 2, we tested whether the items on the compassionate goals scale are empirically separable from the items on each of these other measures by conducting pairs of CFAs. We compared the fit of a model in which compassionate goals items and items from other scales loaded onto the same factor with a model in which compassionate goals items and items from each of the other scales loaded onto separate correlated factors. If the one-factor model provides a better fit to the data, it would suggest that the compassionate goals items assess the same construct as the other measure. If the two-factor model provides a better fit, it would suggest that the compassionate goals scale measures a separate construct. We conducted five sets of CFAs, testing compassionate goals items against each of the other measures of prosocial orientations.

As shown in Table 2, all two-factor models had superior fit compared to the corresponding one-factor models. In addition, all two-factor models resulted in higher confirmatory fit index (CFI) and lower root mean square error of approximation (RMSEA) and χ^2^-to-degrees-of-freedom ratios than their corresponding one-factor models. Thus, items assessing compassionate goals are empirically separable from those assessing compassionate love, communal orientation, communion, unmitigated communion, and empathy.

**TABLE 2:** Study 1 confirmatory factor analyses.

	One-factor solution	Two-factor solution
**Compassionate love and compassionate goals**		
χ^2^	χ^2^ (464) = 2,321.23, *p* < 0.001	χ^2^ (463) = 1,360.72, *p* < 0.001
Change in χ^2^ from one factor to two factors		Δχ^2^ (1) = 960.51, *p* < 0.001
Chi-square mean/degree of freedom (CMIN/DF)	5.00	2.94
Confirmatory fit index (CFI)	0.777	0.892
Root mean square error of approximation (RMSEA)	0.084	0.058
**Communal orientation and compassionate goals**		
χ^2^	χ^2^ (275) = 1,212.04, *p* < 0.001	χ^2^ (274) = 876.96, *p* < 0.001
Change in χ^2^ from one factor to two factors		Δχ^2^ (1) = 335.08, *p* < 0.001
CMIN/DF	4.41	3.20
CFI	0.730	0.826
RMSEA	0.077	0.062
**Communion and compassionate goals**		
χ^2^	χ^2^ (152) = 735.81, *p* < 0.001	χ^2^ (151) = 446.17, *p* < 0.001
Change in χ^2^ from one factor to two factors		Δχ^2^ (1) = 289.64, *p* < 0.001
CMIN/DF	4.84	2.96
CFI	0.803	0.900
RMSEA	0.082	0.059
**Unmitigated communion and compassionate goals**		
χ^2^	χ^2^ (170) = 743.10, *p* < 0.001	χ^2^ (169) = 481.33, *p* < 0.001
Change in χ^2^ from one factor to two factors		Δχ^2^ (1) = 261.77, *p* < 0.001
CMIN/DF	4.37	2.85
CFI	0.781	0.881
RMSEA	0.077	0.057
**Empathy and compassionate goals**		
χ^2^	χ^2^ (135) = 841.66, *p* < 0.001	χ^2^ (134) = 367.09, *p* < 0.001
Change in χ^2^ from one factor to two factors		Δχ^2^ (1) = 474.57, *p* < 0.001
CMIN/DF	6.23	2.74
CFI	0.748	0.917
RMSEA	0.096	0.055

*N = 571 for all analyses.*

#### Do Compassionate Goals Account for Unique Variance in Altruistic Giving to Strangers?

In phase 3 analyses, we tested whether compassionate goals explain variance in altruistic giving to strangers and whether that variance is distinct from that explained by other measures of prosocial orientations. All of the measures of prosocial orientations, including compassionate goals, correlated positively with altruistic giving to strangers and with each other (see Table 1).

To test whether compassionate goals predict unique variance in altruistic giving relative to each of the other measures, we ran five regression equations, each including compassionate goals and one of the other prosocial orientations. Each equation was tested in two steps. In step 1, we regressed altruistic giving on one of the other prosocial orientations; in step 2, we added compassionate goals as a second predictor. This allowed us to determine the increase in variance accounted for by compassionate goals, beyond that accounted for by each of the other prosocial orientations. As shown in the top half of Table 3, each of the other prosocial orientations accounted for significant variance in altruistic giving to strangers in step 1. When we added compassionate goals as an additional predictor in step 2, it accounted for unique variance in altruistic giving to strangers, explaining between 1 and 5% additional variance beyond other prosocial orientations. When we tested each of these models again, adding social desirability and gender as covariates in separate analyses, the associations between compassionate goals and altruistic giving to strangers remained significant (see Supplementary Material and Tables 1, 2).

**TABLE 3:** Study 1 regression analyses predicting altruistic giving to strangers.

	Step 1					Step 2								
	Covariate					Covariate				Compassionate goals				
	*b*	β	95%CI	*p*	*R*^2^	*b*	β	95%CI	*p*	*b*	β	95%CI	*p*	Δ*R*^2^
**Covariates entered in separate analyses**														
Compassionate love	0.42	0.48	[0.36, 0.48]	<0.001	0.23	0.37	0.42	[0.30, 0.44]	<0.001	0.15	0.13	[0.07, 0.25]	0.002	0.01
Communal orientation	0.40	0.30	[0.29, 0.50]	<0.001	0.09	0.24	0.18	[0.12, 0.36]	<0.001	0.28	0.23	[0.17, 0.38]	<0.001	0.04
Communion	0.31	0.27	[0.22, 0.40]	<0.001	0.07	0.13	0.11	[0.03, 0.24]	0.016	0.28	0.24	[0.17, 0.39]	<0.001	0.05
Unmitigated communion	0.38	0.33	[0.29, 0.47]	<0.001	0.11	0.26	0.23	[0.17, 0.36]	<0.001	0.26	0.22	[0.16, 0.36]	<0.001	0.04
Empathic concern	0.29	0.31	[0.22, 0.36)	<0.001	0.09	0.17	0.18	[0.09, 0.25]	<0.001	0.24	0.21	[0.14, 0.35]	<0.001	0.04
**Covariates included in a single analysis**														
Compassionate love	0.39	0.44	[0.30, 0.48]	<0.001	0.24	0.37	0.43	[0.28, 0.46]	<0.001					0.01
Communal orientation	0.05	0.04	[−0.09, 0.20]	0.456		0.03	0.02	[−0.11, 0.17]	0.704					
Communion	0.02	0.01	[−0.10, 0.13]	0.795		−0.02	−0.02	[−0.14, 0.10]	0.767					
Unmitigated communion	0.09	0.08	[−0.01, 0.20]	0.083		0.08	0.07	[−0.03, 0.18]	0.167					
Empathic concern	−0.06	−0.06	[−0.17, 0.05]	0.292		−0.07	−0.07	[−0.17, 0.04]	0.202					
Compassionate goals						0.15	0.13	[0.05, 0.26]	0.005					

*N = 571 for all analyses.*

Because all measures of prosocial orientations correlated positively with each other, we tested a final regression model predicting altruistic giving to strangers, including compassionate love, communal orientation, communion, unmitigated communion, and empathy in step 1, and added compassionate goals in step 2. In step 1, only compassionate love accounted for unique variance in altruistic giving. In step 2, compassionate goals accounted for unique variance in altruistic giving, explaining an additional 1% of variance beyond the other prosocial orientations as a group. The results remained unchanged when we added social desirability and gender as covariates in separate analyses (see Supplementary Material and Tables 1, 2).

### Discussion

The findings of study 1 suggested strong empirical overlap between compassionate goals and compassionate love, communal orientation, communion, unmitigated communion, and empathic concern, although the correlations were not strong enough to indicate that the compassionate goals measure is identical to the other measures. Results from CFAs revealed that items assessing compassionate goals load onto a separate factor from items assessing each of the other prosocial orientations. Thus, compassionate goals are empirically distinct from compassionate love, communal orientation, communion, unmitigated communion, and empathic concern.

These findings also indicate that compassionate goals explain unique variance in altruistic giving to strangers when entered into a regression with each of the other prosocial orientations and when entered with all five other measures simultaneously. Controlling for socially desirable responding and gender did not alter these findings. Thus, although compassionate goals have similarities to other measures of prosocial orientations, they account for variance in altruistic giving to strangers that the other prosocial orientation measures do not.

Whereas study 1 focused on prosocial giving to strangers, study 2 examined whether the unique contribution of compassionate goals to prosocial giving generalizes to giving in close relationships, including to family, friends, and significant others.

## Study 2

Study 2 had three goals. First, we again examined the degree of empirical overlap between compassionate goals and compassionate love, communal orientation, communion, unmitigated communion, and empathic concern. Second, we replicated the CFAs tested in study 1 to determine the distinctiveness of the compassionate goals scale items from items on each of the other measures. Third, we examined whether compassionate goals capture variance in prosocial giving to close others using two measures—social support given to close others and responsiveness to close others—and whether that variance is distinct from that captured by each of the other scales. We again tested whether links between compassionate goals and giving could be accounted for by socially desirable responding and gender.

### Method

#### Participants

Introductory Psychology students (*N* = 318) at a large Midwestern university completed questionnaires in a laboratory session. Fifty-two percent were male, 43% were female, and 5% did not report their gender; their ages ranged from 18 to 23 (*M* = 19.2 years, *SD* = 1.31). The sample was 75% White/Caucasian. We again collected as much data as possible during an academic term. This sample provided adequate power for confirmatory factor analyses (Tabachnick and Fidell, 2013), and *post hoc* power analyses using G^∗^Power (Faul et al., 2007) suggest that this sample provided power of 0.81 to detect small effects (*r* = 0.14) in the correlation and regression analyses described below.

#### Measures

The participants completed measures assessing compassionate goals, compassionate love, communal orientation, communion, unmitigated communion, and empathic concern using the scales described in study 1. They also completed measures of social support given to close others, responsiveness to others, and social desirability. The internal reliabilities (αs) for all study 2 scales appear in Table 4.

**TABLE 4:** Correlations, means, standard deviations, and internal reliabilities for Study 2 variables.

	1.	2.	3.	4.	5.	6.	7.	8.	9.	*M*	*SD*
(1) Compassionate goals	*0.87*									3.89	0.57
(2) Compassionate love	0.61	*0.95*								3.33	0.72
(3) Communal orientation	0.52	0.60	*0.78*							3.62	0.50
(4) Communion	0.51	0.60	0.63	*0.80*						3.88	0.57
(5) Unmitigated communion	0.47	0.60	0.52	0.50	*0.75*					3.21	0.63
(6) Empathic concern	0.49	0.69	0.65	0.64	0.52	*0.82*				3.70	0.68
(7) Social support given	0.47	0.34	0.43	0.44	0.23	0.40	*0.94*			4.26	0.63
(8) Responsiveness	0.58	0.53	0.57	0.62	0.38	0.55	0.64	*0.91*		3.95	0.60
(9) Social desirability	0.23	0.20	0.13	0.20	0.21	0.22	0.01	0.20	*0.71*	0.48	0.15
(10) Gender	0.23	0.21	0.27	0.26	0.14	0.32	0.16	0.21	0.01		

*N = 318. Internal reliabilities are reported on the diagonal in italics. All correlations 0.20 or higher were statistically significant (p ≤ 0.001). The 0.13 correlation between social desirability and communal orientation was also significant (p = 0.024), but the correlation between social desirability and social support given was not significant (p = 0.838). The 0.14 correlation between gender and unmitigated communion was significant (p = 0.020) as was the 0.16 correlation between gender and social support given (p = 0.006); gender was unrelated to social desirability (r = 0.01, p = 0.927). Social desirability scores could range from 0 to 1, with higher scores indicating higher social desirability. Gender was coded such that 1 = male and 2 = female. For all other measures, items were rated on scales from 1 to 5, with higher scores indicating higher levels of the construct.*

Social support given was measured using a modified version of the Multidimensional Survey of Perceived Social Support (Zimet et al., 1988; Dahlem et al., 1991), with items altered to reflect the support that participants give to others. Twelve items were rated on a 1 (very strongly disagree) to 5 (very strongly agree) scale. The sample items include “My friends can talk about their problems with me” and “I really try to help my family.”

Responsiveness to others was measured using the 12-item scale from Cutrona et al. (1997). Twelve items were rated on a scale from 1 (not at all) to 5 (very much). The sample items include “I make others feel valued as people” and “I behave warmly toward others.”

Social desirability was measured using the scale described in Study 1.

### Results

#### Overview of Analyses

We conducted study 2 analyses in three phases. In order to understand the degree of empirical overlap between compassionate goals and compassionate love, communal orientation, communion, unmitigated communion, and empathic concern, in phase 1, we examined the zero-order correlations among compassionate goals and these other measures. In phase 2, we conducted five pairs of CFAs, each comparing the fit of (1) a model in which items from the compassionate goals scale loaded onto the same factor as items from the other measure with (2) a model in which items from the two scales loaded onto separate factors. In phase 3, we tested whether compassionate goals capture variance in giving to close others and whether that variance is distinct from that captured by each of the other scales, social desirability, and gender using the analytic strategy described in study 1.

#### Associations Between Compassionate Goals and Other Prosocial Orientations

First, we examined zero-order correlations among compassionate goals and other prosocial orientations. Replicating the results of study 1, compassionate goals correlated strongly with each of the other prosocial orientations, 0.47 ≤ *r*s ≤ 0.61 (see Table 4).

#### Are Items Assessing Compassionate Goals Scale Empirically Separable From Items Assessing Other Prosocial Orientations?

In phase 2, we sought to replicate the factor structures in study 1 findings, suggesting that the items on the compassionate goals scale are empirically separable from the items on each of these other measures by conducting pairs of CFAs, using the analytical strategy described in study 1.

As shown in Table 5, the findings strongly replicate those of study 1. All two-factor models had superior fit compared to the corresponding one-factor models, and all two-factor models resulted in higher CFI and lower RMSEA and χ^2^-to-degrees-of-freedom ratios than their corresponding one-factor models. Thus, these findings again suggest that items assessing compassionate goals are empirically separable from those assessing compassionate love, communal orientation, communion, unmitigated communion, and empathy.

**TABLE 5:** Study 2 confirmatory factor analyses.

	One-factor solution	Two-factor solution
**Compassionate love and compassionate goals**		
χ^2^	χ^2^ (464) = 1,760.14, *p* < 0.001	χ^2^ (463) = 1,279.96, *p* < 0.001
Change in χ^2^ from one factor to two factors		Δχ^2^ (1) = 480.18, *p* < 0.001
Chi-square mean/degree of freedom (CMIN/DF)	3.79	2.76
Confirmatory fit index (CFI)	0.755	0.846
Root mean square error of approximation (RMSEA)	0.094	0.075
**Communal orientation and compassionate goals**		
χ^2^	χ^2^ (275) = 1,039.662, *p* < 0.001	χ^2^ (274) = 836.78, *p* < 0.001
Change in χ^2^ from one factor to two factors		Δχ^2^ (1) = 202.88, *p* < 0.001
CMIN/DF	3.78	3.05
CFI	0.671	0.758
RMSEA	0.094	0.080
**Communion and compassionate goals**		
χ^2^	χ^2^ (152) = 566.94, *p* < 0.001	χ^2^ (151) = 309.86, *p* < 0.001
Change in χ^2^ from one factor to two factors		Δχ^2^ (1) = 257.08, *p* < 0.001
CMIN/DF	3.73	2.05
CFI	0.788	0.919
RMSEA	0.093	0.058
**Unmitigated communion and compassionate goals**		
χ^2^	χ^2^ (170) = 512.15, *p* < 0.001	χ^2^ (169) = 297.55, *p* < 0.001
Change in χ^2^ from one factor to two factors		Δχ^2^ (1) = 214.60, *p* < 0.001
CMIN/DF	3.01	1.76
CFI	0.802	0.926
RMSEA	0.080	0.049
**Empathy and compassionate goals**		
χ^2^	χ^2^ (135) = 607.32, *p* < 0.001	χ^2^ (134) = 297.01, *p* < 0.001
Change in χ^2^ from one factor to two factors		Δχ^2^ (1) = 310.31, *p* < 0.001
CMIN/DF	4.50	2.22
CFI	0.754	0.915
RMSEA	0.105	0.062

*N = 318 for all analyses.*

#### Do Compassionate Goals Account for Unique Variance in Giving to Close Others?

In phase 3 analyses, we tested whether compassionate goals explain unique variance in giving to close others as indicated by two measures—social support given and responsiveness. All of the measures of prosocial orientations, including compassionate goals, correlated positively with both measures of giving to close others (see Table 4).

We tested whether compassionate goals predict giving to close others independent of each of the other prosocial orientations using the strategy described in study 1. For each giving outcome, we tested five regression equations. Each equation was tested in two steps: In step 1, we regressed the giving outcome on one of the other prosocial orientations; in step 2, we added compassionate goals as a second predictor.

Each of the other prosocial orientations accounted for significant variance in giving social support to close others and responsiveness to close others in step 1 (see Table 6). When we added compassionate goals as a predictor in step 2, it accounted for unique variance in giving to close others, explaining between 8 and 17% additional variance in giving social support to close others and between 10 and 21% additional variance in responsiveness to close others, beyond each of the other prosocial orientations. When we tested each of these models again with social desirability and gender as covariates in separate analyses, the associations between compassionate goals and each outcome remained significant (see Supplementary Material and Tables 3, 4).

**TABLE 6:** Study 2 regression analyses predicting social support given and responsiveness to close others.

	Step 1					Step 2								
	Covariate					Covariate				Compassionate goals				
	*b*	β	95%CI	*p*	*R*^2^	*b*	β	95%CI	*p*	*b*	β	95%CI	*p*	Δ*R*^2^
**DV: social support given**														
**Covariates entered in separate analyses**														
Compassionate love	0.30	0.34	[0.20, 0.40]	<0.001	0.11	0.07	0.08	[−0.05, 0.18]	0.258	0.49	0.43	[0.34, 0.63]	<0.001	0.12
Communal orientation	0.58	0.45	[0.44, 0.72]	<0.001	0.20	0.35	0.27	[0.19, 0.50]	<0.001	0.38	0.34	[0.25, 0.51]	<0.001	0.08
Communion	0.50	0.44	[0.38, 0.62]	<0.001	0.19	0.30	0.26	[0.17, 0.43]	<0.001	0.39	0.35	[0.26, 0.52]	<0.001	0.09
Unmitigated communion	0.25	0.24	[0.13, 0.36]	<0.001	0.06	0.02	0.02	[−0.10, 0.14]	0.702	0.53	0.47	[0.40, 0.66]	<0.001	0.17
Empathic concern	0.39	0.41	[0.29, 0.49]	<0.001	0.17	0.21	0.22	[0.10, 0.32]	<0.001	0.41	0.37	[0.28, 0.54]	<0.001	0.10
**Covariates included in a single analysis**														
Compassionate love	0.01	0.02	[−0.13, 0.15]	0.848	0.24	−0.10	−0.11	[−0.24, 0.04]	0.174					0.07
Communal orientation	0.32	0.25	[0.12, 0.51]	0.002		0.23	0.18	[0.04, 0.42]	0.016					
Communion	0.27	0.24	[0.11, 0.44]	0.001		0.22	0.20	[0.07, 0.38]	0.006					
Unmitigated communion	−0.08	−0.08	[−0.22, 0.05]	0.224		−0.12	−0.12	[−0.25, 0.01]	0.079					
Empathic concern	0.12	0.13	[−0.03, 0.28]	0.119		0.12	0.13	[−0.03, 0.27]	0.104					
Compassionate goals						0.38	0.34	[0.24, 0.53]	<0.001					
**DV: responsiveness**														
**Covariates entered in separate analyses**														
Compassionate love	0.43	0.52	[0.35, 0.52]	<0.001	0.27	0.22	0.26	[0.12, 0.32]	<0.001	0.45	0.42	[0.33, 0.57]	<0.001	0.11
Communal orientation	0.69	0.56	[0.57, 0.81]	<0.001	0.32	0.41	0.35	[0.30, 0.56]	<0.001	0.42	0.40	[0.31, 0.53]	<0.001	0.11
Communion	0.65	0.61	[0.55, 0.75]	<0.001	0.37	0.45	0.42	[0.35, 0.56]	<0.001	0.39	0.37	[0.29, 0.50]	<0.001	0.10
Unmitigated communion	0.36	0.37	[0.26, 0.47]	<0.001	0.14	0.12	0.13	[0.02, 0.23]	0.021	0.55	0.52	[0.44, 0.67]	<0.001	0.21
Empathic concern	0.49	0.55	[0.40, 0.58]	<0.001	0.30	0.30	0.34	[0.21, 0.40]	<0.001	0.43	0.41	[0.32, 0.54]	<0.001	0.13
**Covariates included in a single analysis**														
Compassionate love	0.13	0.15	[0.01, 0.24]	0.028	0.46	0.03	0.04	[−0.09, 0.14]	0.595					0.06
Communal orientation	0.25	0.21	[0.10, 0.41]	0.002		0.18	0.15	[0.03, 0.33]	0.018					
Communion	0.38	0.36	[0.25, 0.51]	<0.001		0.34	0.32	[0.21, 0.47]	<0.001					
Unmitigated communion	−0.06	−0.07	[−0.17, 0.05]	0.266		−0.09	−0.10	[−0.20, 0.01]	0.082					
Empathic concern	0.10	0.12	[−0.02, 0.23]	0.102		0.10	0.12	[−0.01, 0.22]	0.085					
Compassionate goals						0.33	0.31	[0.21, 0.44]	<0.001					

*N = 318 for all analyses.*

Because all measures of prosocial orientations correlated strongly with each other, we tested a final regression model for each giving outcome that included compassionate love, communal orientation, communion, unmitigated communion, and empathy as predictors in step 1 and compassionate goals in step 2. In step 1, communal orientation and communion each accounted for unique variance in both outcomes; compassionate love accounted for additional unique variance in responsiveness. In step 2, compassionate goals accounted for unique variance in both outcomes, explaining an additional 7% of variance in social support given and an additional 6% of variance in responsiveness, beyond the group of prosocial orientations included in step 1. The results remained unchanged when we added social desirability and gender as covariates (see Supplementary Material and Tables 3, 4).

### Discussion

The results from study 2 replicate those of study 1. Compassionate goals correlated strongly with other measures of prosocial orientations but not strongly enough to suggest that they assess identical constructs. Additionally, the results from CFAs suggested that items assessing compassionate goals are distinct from items assessing each of the other prosocial orientations. Although compassionate love, communal orientation, communion, unmitigated communion, and empathic concern all correlated with both giving social support and responsiveness to close others, compassionate goals predicted these giving outcomes beyond each of the other prosocial orientations entered alone or together as a group. These findings lend additional support to the proposition that compassionate goals account for aspects of giving to others that cannot be accounted for by other measures of prosocial orientations. Social desirability and gender did not account for associations between compassionate goals and giving.

Studies 1 and 2 assessed the unique contribution of compassionate goals in predicting giving to people in general. In study 3, we examined whether compassionate goals uniquely predict giving to specific individuals, namely, friends. Whereas studies 1 and 2 focused on predicting *whether* people give to others, study 3 also investigated *why* people give to others. Specifically, study 3 tested whether compassionate goals uniquely predict other-focused reasons for giving to specific friends, controlling for self-focused reasons for giving. Because compassionate goals reflect intentions to support others and not harm them, we hypothesized that compassionate goals would be uniquely related to other-focused reasons for giving and that other-focused reasons for giving predict friends’ gratitude. In exploratory analyses, we also tested whether people with compassionate goals feel more gratitude toward their friends.

## Study 3

Study 3 had three goals. First, as in studies 1 and 2, we examined the degree of empirical overlap between compassionate goals and the other measures of prosocial orientations. Second, we tested whether compassionate goals explain variance in other-focused reasons for giving and gratitude toward friends and whether the variance explained by compassionate goals is distinct from that explained by the other prosocial orientations. Additionally, we examined whether links between compassionate goals and reasons for giving and gratitude could be accounted for by gender. Finally, we examined whether compassionate goals indirectly predict others’ gratitude through their other-focused reasons for giving and whether the strength of these associations depended on relationship length.

### Method

#### Participants

Ninety-nine same-sex pairs (total *N* = 198; 81% female) were recruited in public spaces at a large Midwestern university. Same-sex pairs sitting together were invited to participate in a 15-min survey on friendships. The participants received a $5 gift card to a local ice cream shop.

The participants had known their friends for between a few days and 20 years (*M* = 3.59 years, *SD* = 4.69); their ages ranged from 18 to 33 years (*M* = 19.92 years, *SD* = 1.71). The sample was 85% White/Caucasian. The sample size was determined by available funding. *Post hoc* power analyses using APIMPower (Ackerman and Kenny, 2016, December) suggest that this sample provided 0.804 power to detect actor and partner effects of 0.19.

#### Measures

The participants completed measures of compassionate goals, compassionate love, communal orientation, communion, unmitigated communion, and empathic concern using the scales described in study 1. They also reported their reasons for giving to their friends and gratitude toward friends. The participants completed the surveys independent of their friends. The internal reliabilities for all study 3 scales are reported in Table 7.

**TABLE 7:** Intraclass correlations, means, standard deviations, and internal reliabilities for study 3 variables.

	1	2	3	4	5	6	7	8	9	*M*	*SD*
(1) Compassionate goals	*0.84*									4.05	0.50
(2) Compassionate love	0.63***	*0.95*								3.43	0.69
(3) Communal orientation	0.39***	0.40***	*0.76*							3.76	0.48
(4) Communion	0.55***	0.52***	0.55***	*0.71*						3.99	0.49
(5) Unmitigated communion	0.50***	0.56***	0.42***	0.55***	*0.68*					3.40	0.56
(6) Empathic concern	0.52***	0.60***	0.55***	0.66***	0.52***	*0.75*				3.83	0.56
(7) Other-focused reasons for giving	0.40***	0.28***	0.16*	0.25***	0.28***	0.32***	*0.79*			4.12	0.61
(8) Self-focused reasons for giving	−0.00	−0.01	−0.13	−0.01	0.06	−0.05	0.26**	0.84		2.88	0.85
(9) Gratitude	0.43***	0.28***	0.22**	0.40***	0.18*	0.37***	0.52***	0.02	*0.78*	4.49	0.53
(10) Gender	0.07	−0.02	0.14*	0.09	0.02	0.13	0.24**	0.05	0.27***		

N = 198. Internal reliabilities are reported on the diagonal in italics. Gender was coded such that 1 = male and 2 = female. All other measures were rated on scales from 1 to 5, with higher scores indicating higher levels of the construct. *p < 0.05, **p < 0.01, ***p < 0.001.

Reasons for giving to friends were assessed using a two-part measure created for this research. The participants described “How you try to be helpful, generous, thoughtful, or supportive of ___. Include what you do, why you do it, and how often you do those things.” They then rated “The importance of each of the following reasons for your decision to do things for ___” on a scale from 1 (not at all important) to 5 (extremely important). Seven items assessed other-focused reasons for giving. The sample items include “Because I did not want my friend to be hurt” and “Because I thought it would be helpful or supportive for ___.” Seven items assessed self-focused reasons for giving. The sample items include “To show ___ that I am a good person” and “To prevent my friend from getting angry at me.”

Gratitude toward friends was also assessed using a two-part measure created for this investigation. The participants described “how ___ is helpful, generous, thoughtful, or supportive of you. Include what s/he does, how it makes you feel, and how often ____ does those things for you.” Then, they received these instructions: “People often feel different things in different situations. When ___ does things for you, how much do you feel?” followed by 17 adjectives rated on a scale from 1 (not at all) to 5 (extremely). Gratitude was assessed with three items: “thankful,” “grateful,” and “appreciative” (Emmons and McCullough, 2003).

### Results

#### Overview of Analyses

We conducted study 3 analyses in three phases. In phase 1, we examined the zero-order correlations between compassionate goals and the measures of prosocial orientations included in studies 1 and 2. In phase 2, we tested whether compassionate goals capture variance in other-focused reasons for giving to friends and gratitude toward friends that is unique from that captured by other measures of prosocial orientations, again using the general analytic approach described in study 1. We also tested whether associations between compassionate goals and reasons for giving and gratitude could be accounted for by gender or were moderated by relationship length. In phase 3, we tested a path model in which the participants’ compassionate goals predicted their reasons for giving to friends, which in turn led to friends’ gratitude toward the participants.

#### Associations Between Compassionate Goals and Other Prosocial Orientations

As in studies 1 and 2, we examined the bivariate associations between compassionate goals and other prosocial orientations. Table 7 shows the means, the standards deviations, and the internal reliabilities. It also shows the intrapersonal (i.e., within-person) intraclass correlations, which adjust for the degree of non-independence between dyad members (Griffin and Gonzalez, 1995) for all study 3 variables. As in studies 1 and 2, compassionate goals correlated strongly with each of the other prosocial orientations, with intraclass correlations ranging between 0.39 and 0.63.

#### Do Compassionate Goals Account for Unique Variance in Other-Focused Reasons for Giving and Gratitude?

In phase 2 analyses, we tested whether compassionate goals explain unique variance in other-focused giving and gratitude. As shown in Table 7, compassionate goals and all other measures of prosocial orientations correlated positively with both of these outcomes.

Because individuals were nested within dyads, we used the MIXED command in SPSS to account for the non-independence of individuals within dyads; all predictors were centered. As in studies 1 and 2, we tested five regression equations for each outcome. Each equation was tested in two steps: In step 1, we regressed the outcome on one of the other prosocial orientations; in step 2, we added compassionate goals as a second predictor. In the analyses predicting other-focused reasons for giving, we controlled for self-focused reasons for giving.

Each of the other prosocial orientations accounted for significant variance in other-focused reasons for giving (Table 8) and own gratitude (Table 9) in step 1, with one exception: communal orientation did not predict other-focused reasons for giving. When we added compassionate goals as an additional predictor in step 2, it accounted for between 9 and 13% additional variance in other-focused reasons for giving and between 6 and 16% additional variance in own gratitude, beyond each of the other prosocial orientations. When we tested each of these models again, adding gender as a covariate, the associations between compassionate goals and other-focused reasons for giving and gratitude remained significant (see Supplementary Material and Table 5).

**TABLE 8:** Study 3 analyses predicting other-focused reasons for giving.

	Model 1					Model 2				
	*b*	95%CI	*pr*	*p*	*Pseudo*-Δ*R*^2^	*b*	95%CI	*pr*	*p*	*Pseudo*-Δ*R*^2^
Self-focused reasons for giving	0.15	[0.06, 0.24]	0.24	0.001	0.08	0.16	[0.07, 0.25]	0.25	<0.001	0.09
Compassionate love	0.22	[0.11, 0.34]	0.27	<0.001		0.05	[−0.09, 0.19]	0.05	0.458	
Compassionate goals						0.40	[0.20, 0.59]	0.28	<0.001	
Self-focused reasons for giving	0.17	[0.07, 0.26]	0.25	0.001	0.03	0.16	[0.07, 0.24]	0.24	0.001	0.13
Communal orientation	0.14	[−0.03, 0.31]	0.12	0.097		−0.01	[−0.18, 0.16]	−0.01	0.930	
Compassionate goals						0.44	[0.28, 0.61]	0.36	<0.001	
Self-focused reasons for giving	0.16	[0.07, 0.26]	0.24	0.001	0.06	0.16	[0.07, 0.25]	0.25	<0.001	0.11
Communion	0.21	[0.05, 0.37]	0.19	0.011		−0.02	[−0.20, 0.15]	−0.02	0.811	
Compassionate goals						0.45	[0.27, 0.63]	0.34	<0.001	
Self-focused reasons for giving	0.15	[0.06, 0.24]	0.23	0.001	0.07	0.16	[0.07, 0.25]	0.25	<0.001	0.11
Unmitigated communion	0.23	[0.10, 0.37]	0.24	0.001		0.08	[−0.07, 0.23]	0.08	0.296	
Compassionate goals						0.40	[0.23, 0.58]	0.31	<0.001	
Self-focused reasons for giving	0.18	[0.08, 0.26]	0.27	<0.001	0.11	0.17	[0.08.26]	0.27	<0.001	0.08
Empathic concern	0.30	[0.16, 0.45]	0.29	<0.001		0.14	[−0.02, 0.30]	0.12	0.087	
Compassionate goals						0.37	[0.19, 0.55]	0.28	<0.001	
Self-focused reasons for giving	0.15	[0.05, 0.24]	0.22	0.002	0.10	0.15	[0.06, 0.24]	0.24	0.001	0.07
Compassionate love	0.12	[−0.03, 0.27]	0.12	0.105		0.00	[−0.15, 0.16]	0.00	0.954	
Communal orientation	−0.07	[−0.27, 0.13]	−0.05	0.480		−0.07	[−0.26, 0.13]	−0.05	0.500	
Communion	−0.08	[−0.29, 0.14]	−0.06	0.491		−0.13	[−0.35, 0.08]	−0.10	0.224	
Unmitigated communion	0.12	[−0.06, 0.29]	0.11	0.184		0.08	[−0.09, 0.26]	0.08	0.337	
Empathic concern	0.22	[0.01, 0.43]	0.15	0.041		0.19	[−0.02, 0.39]	0.13	0.070	
Compassionate goals						0.38	[0.17, 0.58]	0.25	<0.001	

*Our calculated percent of variance was accounted for in outcomes using pseudo-ΔR^2^ (Kenny et al., 2006). In step 1, pseudo-ΔR^2^ reflects the additional variance explained by the prosocial orientation measure included in that model. Pseudo-ΔR^2^ in model 1 was calculated by comparing model 1 to a model including only self-focused reasons for giving as the predictor. In step 2, pseudo-ΔR^2^ reflects the additional variance explained when compassionate goals are added to the model.*

**TABLE 9:** Study 3 analyses predicting own gratitude.

	Model 1					Model 2				
	*b*	95%CI	*pr*	*p*	*Pseudo*-Δ*R*^2^	*b*	95%CI	*pr*	*p*	*Pseudo*-Δ*R*^2^
Compassionate love	0.21	[0.11, 0.31]	0.29	<0.001	0.07	0.03	[−0.09, 0.16]	0.04	0.576	0.11
Compassionate goals						0.40	[0.23, 0.57]	0.31	<0.001	
Communal orientation	0.18	[0.03, 0.32]	0.17	0.015	0.08	0.04	[−0.11, 0.18]	0.04	0.609	0.14
Compassionate goals						0.41	[0.27, 0.55]	0.39	<0.001	
Communion	0.38	[0.25, 0.51]	0.39	<0.001	0.15	0.22	[0.08, 0.37]	0.23	0.003	0.07
Compassionate goals						0.32	[0.16, 0.47]	0.29	<0.001	
Unmitigated communion	0.13	[0.01, 0.26]	0.16	0.035	0.03	−0.04	[−0.17, 0.09]	−0.05	0.517	0.16
Compassionate goals						0.45	[0.30, 0.61]	0.39	<0.001	
Empathic concern	0.31	[0.18, 0.43]	0.33	<0.001	0.13	0.14	[0.00, 0.28]	0.15	0.043	0.08
Compassionate goals						0.35	[0.20, 0.51]	0.30	<0.001	
Compassionate love	0.10	[−0.02, 0.22]	0.12	0.111	0.20	0.00	[−0.13, 0.13]	0.00	0.955	0.06
Communal orientation	−0.07	[−0.24, 0.09]	−0.07	0.380		−0.07	[−0.23, 0.09]	−0.07	0.367	
Communion	0.29	[0.11, 0.47]	0.26	0.002		0.24	[0.06, 0.42]	0.22	0.008	
Unmitigated communion	−0.08	[−0.22, 0.07]	−0.08	0.316		−0.10	[−0.24, 0.04]	−0.11	0.158	
Empathic concern	0.13	[−0.04, 0.31]	0.11	0.138		0.10	[−0.07, 0.27]	0.09	0.238	
Compassionate goals						0.32	[0.14, 0.49]	0.25	<0.001	

*Our calculated percent of variance was accounted for in outcomes using pseudo-ΔR^2^ (Kenny et al., 2006). In step 1, pseudo-ΔR^2^ reflects the additional variance explained by the prosocial orientation measure included in that model. Pseudo-ΔR^2^ in model 1 was calculated by comparing model 1 to a model including no predictor. In step 2, pseudo-ΔR^2^ reflects the additional variance explained when compassionate goals are added to the model.*

Because all measures of prosocial orientations correlated strongly with each other (see Table 7), we tested a final model for both outcomes, each including compassionate love, communal orientation, communion, unmitigated communion, and empathy in step 1 and adding compassionate goals in step 2. In step 1, empathic concern accounted for unique variance in other-focused reasons for giving (see Table 8) and communion accounted for unique variance in own gratitude (see Table 9). In step 2, compassionate goals accounted for unique variance in both outcomes, explaining an additional 7% of variance in other-focused reasons for giving and an additional 6% of variance in own gratitude, beyond that explained by the other measures of prosocial orientations. The results remained unchanged when we added gender as a covariate (see Supplementary Material and Table 5).

#### Compassionate Goals, Other-Focused Reasons for Giving, and Friends’ Gratitude

Finally, we tested whether the participants’ compassionate goals indirectly predicted friends’ gratitude through the participants’ other-focused reasons for giving. Specifically, we tested a path model using a multiple regression strategy in which the participants’ compassionate goals predicted their other-focused reasons for giving, which then predicted friends’ gratitude toward the participants. [We did not use MEDYAD (Coutts et al., 2019) to test mediation because MEDYAD requires distinguishable dyads]. When we regressed the participants’ other-focused reasons for giving on their compassionate goals, controlling for their self-focused reasons for giving, the participants’ compassionate goals predicted their greater other-focused reasons for giving, *b* = 0.44, *SE* = 0.08, *t*(193.86) = 5.71, 95%CI [0.29, 0.60], *p* < 0.001, pr = 0.38. When we regressed friends’ gratitude on the participants’ other-focused reasons for giving and compassionate goals controlling for their self-focused reasons for giving, the participants’ friend-centered reasons for giving predicted friends’ gratitude, *b* = 0.30, *SE* = 0.07, *t*(184.68) = 4.52, 95%CI [0.17, 0.43], *p* < 0.001, pr = 0.32. We calculated the 95% confidence interval for the indirect effect using the Monte Carlo method for assessing mediation (Selig and Preacher, 2008). This confidence interval did not include zero, 95%CI [0.07, 0.21], indicating a significant indirect effect of compassionate goals on friends’ gratitude through the participants’ other-focused reasons for giving. The results did not change when we controlled for the other measures of prosocial orientations (see Supplementary Material and Table 6), and relationship length did not moderate the association between the participants’ compassionate goals and their other-focused reasons for giving [*t*(187.51) = 0.67, *pseudo*-Δ*R*^2^ = 0.00, *p* = 0.502] or the association between the participants’ other-focused reasons for giving and friend’s gratitude [*t*(179.62) = −0.64, *pseudo*-Δ*R*^2^ = 0.00, *p* = 0.643]. We also considered an alternative model in which friends’ gratitude predicted the participants’ compassionate goals, which in turn predicted the participants’ other-focused reasons for gratitude; however, friends’ gratitude did not directly predict the participants’ compassionate goals, *b* = 0.08, *SE* = 0.07, *t*(189.08) = 1.25, 95%CI [−0.05, 0.22], *p* = 0.213, pr = 0.09.

### Discussion

Study 3 replicated the findings of studies 1 and 2 that compassionate goals overlap with other measures of prosocial orientations. However, this overlap again is not sufficiently strong to suggest that they assess identical constructs.

Study 3 also found that compassionate goals account for unique variance in other-focused reasons for giving and feelings of gratitude toward friends, controlling for other prosocial orientations. Although other measures of prosocial orientations were generally positively associated with other-focused reasons for giving and gratitude, compassionate goals predicted each of these outcomes even with the other measures controlled. Similar to studies 1 and 2, compassionate goals also explained variance in other-focused reasons for giving and feelings of gratitude distinct from that explained by the other prosocial orientations when considered as a group. Gender did not account for these associations. Thus, compassionate goals account for aspects of other-focused reasons for giving and gratitude that cannot be accounted for by other measures of prosocial orientations.

Finally, the results of study 3 supported a path model in which the participants’ intentions to be supportive indirectly predict friends’ greater gratitude through the participants’ more friend-centered reasons for giving. Friends appear to be sensitive to the participants’ reasons for giving—when participants gave for friend-centered reasons, friends expressed more gratitude. These effects did not differ for newer compared to more seasoned relationships.

## General Discussion

Based on their theory of egosystem and ecosystem social motivation (Crocker and Canevello, 2015, 2017), Crocker and Canevello (2008) developed the compassionate goals scale as an indicator of ecosystem motivation. This measure, which assesses intentions to be supportive and constructive and not harm others, has demonstrated reliability and both construct and predictive validity in numerous investigations conducted over the past decade (e.g., Crocker and Canevello, 2012, 2015, 2017). However, to date, it has been unclear what the compassionate goals scale adds to research on prosocial orientation beyond existing measures. Specifically, research has not established whether the compassionate goals measure is empirically distinct from existing measures of prosocial orientations and whether it predicts unique variance in indicators of prosocial behavior. The present studies address this gap.

The results from three studies suggest that the compassionate goals scale is empirically distinct from and adds predictive power beyond five commonly used existing measures of prosocial orientations—compassionate love (Fehr and Sprecher, 2008), communal orientation (Clark et al., 1987), communion (Spence et al., 1979), unmitigated communion (Fritz and Helgeson, 1998), and empathic concern (Davis, 1983). Across studies, compassionate goals correlated with these existing measures, sharing approximately 25% of variance with each of them. However, CFAs in studies 1 and 2 demonstrated that, when tested against each of the existing measures, the items in the compassionate goals scale were empirically distinct from the items in each of the other measures. When we examined the fit of items from the compassionate goals measure with items from each of the other measures, two-factor models, in which compassionate goal items loaded on their own factor, fit the data reasonably well and fit the data better than models in which the compassionate goals items loaded on the same factor as the items on each of the other measures.

We also examined the zero-order correlations between all six measures of prosocial orientations and self-reported giving to strangers and close others and prosocial reasons for giving. Across studies, all measures of prosocial orientations correlated positively with these outcomes, raising concerns about whether the compassionate goals scale is redundant with existing measures of prosocial orientations. However, the regression analyses suggested that this was not the case. When we paired the compassionate goals measure with each of the other prosocial orientation measures in individual regression equations, the compassionate goals measure accounted for unique variance in outcomes beyond the other measures in each study. Furthermore, when we entered all six prosocial orientation measures together in regression models, compassionate goals explained significant unique variance in all giving outcomes in every study. Thus, the compassionate goals scale is both empirically distinct from existing measures and adds predictive value beyond the existing measures included in these studies. Notably, compassionate goals captured seemingly greater proportions of unique variance in giving to close others (studies 2 and 3), compared to strangers (study 1). The egosystem–ecosystem theory does not assume that the effects of compassionate goals differ by target or relationship type, although the mean levels of compassionate goals do differ by relationship type. However, these findings are consistent with existing work suggesting that prosocial orientations more strongly predict giving in the context of close others (e.g., Clark and Mills, 1993; Stürmer et al., 2005).

These results are not simply due to the general tendency to respond in socially desirable ways or gender. Consistent with previous research (e.g., Crocker and Canevello, 2008), compassionate goals correlated positively with social desirability in studies 1 and 2. Women reported greater compassionate goals in studies 1 and 2 greater other-focused reasons for giving and gratitude in study 3. However, the associations between compassionate goals and giving remained significant when we controlled for these variables, suggesting that the links between compassionate goals and giving are not simply due to a desire to present the self in socially acceptable ways or gender. Additionally, associations between the participants’ compassionate goals and their other-focused reasons for giving and between the participants’ other-focused reasons for giving and friend’s gratitude did not depend on relationship length, suggesting that the interpersonal prosocial processes associated with compassionate goals are not different in newer *versus* older relationships.

These findings provide consistent evidence that the compassionate goals measure captures a unique aspect of prosocial orientations relative to other well-established and commonly used measures from this literature. What exactly makes the compassionate goals measure distinct? The compassionate goals scale was designed to assess ecosystem motivations for social behavior. Consistent with the theory of egosystem and ecosystem motivation (Crocker and Canevello, 2015, 2017), items in the compassionate goals scale explicitly assess people’s intentions to be supportive and constructive and to not harm others. In contrast, existing measures of prosocial orientations, including compassionate love, communal orientation, communion, unmitigated communion, and empathic concern, do not directly or explicitly assess goals to be supportive and constructive and not harm others. Thus, the unique contribution of the compassionate goals scale appears to be its focus on *intentions* to be supportive and not harmful.

The findings of study 3 for other-focused reasons for giving support this interpretation. Each of the measures of prosocial orientations in this investigation, with the exception of communal orientation, predicted friend-centered reasons for giving when entered into separate regressions that included self-centered reasons for giving as a covariate. However, when we added compassionate goals as a predictor to each of the models, none of those associations remained significant, suggesting that compassionate goals account for associations between the other prosocial orientations and friend-centered reasons for giving. Furthermore, the compassionate goals measure significantly predicted other-centered reasons for giving to close others even when controlling for each of the other measures in separate regressions and when controlling for all of them simultaneously. Thus, other-centered reasons for giving relate uniquely to the compassionate goals scale and were unrelated to the other prosocial orientations assessed in this investigation. To the extent that the other measures predict other-centered reasons, it is due to their shared variance with the compassionate goals scale.

People with compassionate goals not only give more to others—they also feel more grateful for what they receive from others. The finding that people with higher compassionate goals feel more gratitude toward their friends is consistent with evidence that they view giving to others as working in nonzero-sum ways (Crocker et al., 2017b). People with compassionate goals do not view giving to others as costly to themselves. They rather seem to recognize that they also receive from their friends and feel grateful in return. Future research should investigate whether people with compassionate goals feel grateful because they receive more from others or because they are more appreciative of what they receive.

Study 3 also examined the downstream consequences of compassionate goals and other-focused reasons for giving on friends’ gratitude. Compassionate goals specifically assess the intentions to be supportive and constructive and to not harm others. Intentions are an important aspect of prosocial orientations because others are sensitive to people’s intentions (e.g., Feeney and Collins, 2003). In study 3, friends reported more gratitude when others gave to them in order to support and not harm them, indicating that compassionate goals matter not just for people’s own reasons for giving but also for others’ responses to receiving. When people have compassionate goals, they give for other-centered reasons, which inspire more gratitude in others.

We do not claim that the compassionate goals scales should supersede other measures of prosocial orientations. Our point rather is that the compassionate goals scale, which was developed to assess intentions to be constructive and supportive, adds something unique to the literature on prosocial motivation that existing measures do not capture.

### Caveats

It is possible that our results would differ if we had included other measures of prosocial orientations. We choose five prosocial orientation measures that are commonly used in the literature, have demonstrated associations with prosocial behaviors, and that assess several components of prosocial orientations. It is also possible that our results would differ if we had measured other outcomes such as well-being, relationship quality, or physical health. Accordingly, we view the current investigation as an initial attempt to pinpoint the relative contribution of the compassionate goals measure to the prosociality literature. Future work including other measures of prosocial orientations and other outcomes is warranted.

In addition, these studies do not include behavioral measures. However, in study 3, we included pairs of friends who reported on their own prosocial orientations, reasons for giving, and feelings of gratitude and their gratitude toward their friend. The participants’ compassionate goals predicted their other-focused reasons for giving, which in turn predicted their friend’s gratitude, suggesting that compassionate goals shape the experiences of relationship partners. Thus, other-centered intentions likely have some behavioral expressions that relationship partners can detect and perhaps report on.

Finally, these samples consisted primarily of 18–21-year-old college students, and the study 3 participants were predominantly female, which limits the generalizability of these findings. While we do not expect a different pattern of results in more diverse groups, future research should test these associations in older, non-student samples.

### Conclusion

The present investigation examines the relative contribution of the compassionate goals measure, which assesses ecosystem motivation through intentions to be supportive and constructive and not harm others. Although the compassionate goals measure correlates with well-established measures of prosocial orientations, it also captures unique variance in giving and other-focused reasons for giving and has unique downstream implications for others’ gratitude. Thus, the compassionate goals measure appears to make a unique and significant contribution to our understanding of prosociality.

## Data Availability Statement

The data and syntax can be found at https://osf.io/gxyf7/?view_only=3214297b709344c380b92577ae17167e or can be obtained by request from the corresponding author.

## Ethics Statement

The studies involving human participants were reviewed and approved by Institutional Review Board, The Ohio State University. The patients/participants provided their written informed consent to participate in this study.

## Author Contributions

AC and JC shared in the conception, design, and crafting of the final version of this work. AC conducted data analyses. JC provided resources to conduct the research and supervised the projects. AC and JC were jointly accountable for the content of the work, ensuring that all aspects related to the accuracy or the integrity of the study are investigated and resolved in an appropriate way. Both authors contributed to the article and approved the submitted version.

## Conflict of Interest

The authors declare that the research was conducted in the absence of any commercial or financial relationships that could be construed as a potential conflict of interest.
