# Supplementary material for: Prosocial Orientations: Distinguishing Compassionate Goals From Other Constructs
Source: Front Psychol. 2020 Sep 17;11:538165. doi: 10.3389/fpsyg.2020.538165 (PMC7527500; doi:10.3389/fpsyg.2020.538165)
Supplement: Supplementary file 1 [file Data_Sheet_1.PDF]

Table of Contents:

1. Supplemental Table 1: page 2
    a. Study 1 Regression Analyses Predicting Altruistic Giving to Strangers, Controlling for Social Desirability; referenced in section 2.2.4

2. Supplemental Table 2: page 3
    a. Study 1 Regression Analyses Predicting Altruistic Giving to Strangers, Controlling for Gender; referenced in section 2.2.4

3. Supplemental Table 3: pages 4-5
    a. Study 2 Regression Analyses Predicting Social Support Given and Responsiveness to Close Others, Controlling for Social Desirability; referenced in section 3.2.3

4. Supplemental Table 4: pages 6-7
    a. Study 2 Regression Analyses Predicting Social Support Given and Responsiveness to Close Others, Controlling for Gender; referenced in section 3.2.3

5. Supplemental Table 5: pages 8-9
    a. Study 3 Analyses Predicting Other-Focused Reasons for Giving and Gratitude, Controlling for Gender; referenced in section 4.2.3

6. Supplement to Section 4.2.4 Compassionate Goals, Other-Focused Reasons for Giving, and Friend's Gratitude: page 10

7. Supplemental Table 6: page 10
    a. Study 3 Supplemental Analyses Examining Participants' Other-Focused Reasons for Giving, Controlling for Participants' Compassionate Goals, Self-Focused Reasons for Giving, and Other Measures of Prosocial Orientations; referenced in section 4.2.4

8. Measures for All Studies: pages 11-20

Supplemental Table 1.

Study 1 Regression Analyses Predicting Altruistic Giving to Strangers, Controlling for Social Desirability

	Covariate				Social Desirability				Compassionate Goals			
	b	β	95%CI	*p*	b	β	95%CI	*p*	b	β	95%CI	*p*
Covariates Entered in Separate Analyses												
Compassionate Love	.36	.41	[.28, .43]	< .001	.21	.05	[-.12, .54]	.208	.14	.12	[.05, .24]	.004
Communal Orientation	.23	.17	[.11, .34]	< .001	.52	.12	[.17, .87]	.003	.24	.21	[.13, .35]	< .001
Communion	.13	.11	[.03, .24]	.016	.49	.11	[.14, .85]	.006	.28	.34	[.17, .39]	< .001
Unmitigated Communion	.25	.22	[.16, .35]	< .001	.51	.12	[.16, .85]	.004	.23	.19	[.13, .33]	< .001
Empathic Concern	.17	.18	[.09, .25]	< .001	.51	.12	[.16, .85]	.004	.24	.21	[.14, .35]	< .001
Covariates Entered in a Single Analysis												
Compassionate Love	.36	.41	[.27, .45]	< .001								
Communal Orientation	.03	.02	[-.03, .19]	.661								
Communion	-.03	-.03	[-.15, .09]	.611								
Unmitigated Communion	.08	.07	[-.03, .09]	.153								
Empathic Concern	-.06	-.07	[-.17, .04]	.223								
Social Desirability	.22	.05	[-.11, .56]	.190								
Compassionate Goals	.14	.12	[.04, .25]	.009								

N = 571 for all analyses

Supplemental Table 2.

Study 1 Regression Analyses Predicting Altruistic Giving to Strangers, Controlling for Gender

	Covariate				Gender				Compassionate Goals			
	b	β	95%CI	*p*	b	β	95%CI	*p*	b	β	95%CI	*p*
Covariates Entered in Separate Analyses												
Compassionate Love	.38	.43	[.30, .45]	< .001	-.08	-.06	[-.17, .02]	.116	.16	.14	[.07, .26]	.001
Communal Orientation	.24	.19	[.13, .36]	< .001	-.03	-.02	[-.13, .08]	.616	.28	.24	[.17, .39]	< .001
Communion	.16	.14	[.05, .26]	.004	-.01	-.01	[-.11, .10]	.886	.31	.26	[.20, .41]	< .001
Unmitigated Communion	.26	.23	[.17, .36]	< .001	-.01	-.01	[-.11, .09]	.836	.27	.23	[.16, .37]	< .001
Empathic Concern	.19	.20	[.11, .28]	< .001	-.05	-.04	[-.16, .05]	.318	.28	.20	[.18, .38]	< .001
Covariates Entered in a Single Analysis												
Compassionate Love	.38	.43	[.29, .47]	< .001								
Communal Orientation	.03	.02	[-.11, .17]	.660								
Communion	-.02	-.02	[-.14, .10]	.705								
Unmitigated Communion	.07	.06	[-.04, .18]	.186								
Empathic Concern	-.05	-.06	[-.16, .05]	.315								
Gender	-.07	-.05	[-.17, .03]	.187								
Compassionate Goals	.16	.13	[.05, .27]	.004								

N = 571 for all analyses

Supplemental Table 3.

Study 2 Regression Analyses Predicting Social Support Given and Responsiveness to Close Others, Controlling for Social Desirability

	Covariate				Social Desirability				Compassionate Goals			
	b	β	95%CI	*p*	b	β	95%CI	*p*	b	β	95%CI	*p*
DV: Social Support Given												
Covariates Entered in Separate Analyses												
Compassionate Love	.07	.08	[-.04, .19]	.207	-.46	-.11	[-.92, .00]	.051	.51	.45	[.36, .66]	< .001
Communal Orientation	.35	.27	[.19, .50]	< .001	-.43	-.10	[-.87, .02]	.060	.40	.36	[.27, .54]	< .001
Communion	.32	.28	[.19, .45]	< .001	-.56	-.13	[-1.01, -.12]	.013	.41	.37	[.28, .54]	< 001
Unmitigated Communion	.04	.04	[-.08, .16]	> .250	-.45	-.10	[-.92, .01]	.055	.55	.49	[.41, .68]	< .001
Empathic Concern	.23	.24	[.12, .34]	< .001	-.56	-.13	[-1.01, -.11]	.015	.43	.39	[.30, .57]	< .001
Covariates Entered in a Single Analysis												
Compassionate Love	-.10	-.11	[-.24, .04]	.149								
Communal Orientation	.21	.16	[.02, .40]	.031								
Communion	.24	.21	[.08, .40]	.003								
Unmitigated Communion	-.11	-.10	[-.24, .03]	.111								
Empathic Concern	.14	.15	[-.01, .29]	.059								
Social Desirability	-.53	-.12	[-.97, -.08]	.021								
Compassionate Goals	.40	.36	[.26, .55]	< .001								
DV: Responsiveness												
Covariates Entered in Separate Analyses												
Compassionate Love	.21	.26	[.12, .31]	< .001	.21	.05	[-.18, .61]	>.250	.44	.42	[.31, .56]	< .001
Communal Orientation	.43	.35	[.30, .56]	< .001	.31	.08	[-.07, .68]	.108	.10	.38	[.29, .51]	< .001
Communion	.45	.42	[.34, .56]	< .001	.10	.03	[-.26, .47]	>.250	.39	.37	[.28, .49]	< .001
Unmitigated Communion	.12	.12	[.01, .22]	.030	.22	.06	[-.18, .63]	>.250	.54	.51	[.43, .66]	< .001
Empathic Concern	.30	.33	[.21, .39]	< .001	.12	.03	[-.27, .50]	>.250	.43	.41	[.32, .54]	< .001
Covariates Entered in a Single Analysis												
Compassionate Love	.03	.04	[-.08, .14]	>.250								
Communal Orientation	.19	.16	[.04, .34]	.014								
Communion	.34	.32	[.21, .47]	<.001								
Unmitigated Communion	-.10	-.10	[-.20, .01]	.072								
Empathic Concern	.10	.11	[-.02, .22]	.106								
Social Desirability	.16	.04	[-.20, .51]	>.250								

Compassionate Goals	.32	.30	[.20, .44]	<.001

N = 318 for all analyses

Supplemental Table 4.

Study 2 Regression Analyses Predicting Social Support Given and Responsiveness to Close Others, Controlling for Gender

	Covariate				Gender				Compassionate Goals			
	b	β	95%CI	*p*	b	β	95%CI	*p*	b	β	95%CI	*p*
DV: Social Support Given												
Covariates Entered in Separate Analyses												
Compassionate Love	.07	.08	[-.04, .18]	.323	.06	.05	[-.08, .19]	.392	.47	.41	[.32, .61]	< .001
Communal Orientation	.35	.27	[.20, .50]	< .001	.01	.01	[-.12, .14]	.889	.37	.32	[.23, .50]	< .001
Communion	.31	.27	[.18, .43]	< .001	.01	.01	[-.12, .15]	.833	.38	.34	[.25, .51]	< .001
Unmitigated Communion	.01	.01	[-.11, .13]	.872	.07	.05	[-.07, .20]	.322	.51	.45	[.38, .65]	< .001
Empathic Concern	.21	.22	[.10, .32]	< .001	.01	.01	[-.13, .14]	.901	.41	.36	[.28, .54]	< .001
Covariates Entered in a Single Analysis												
Compassionate Love	-.10	-.11	[-.24, .04]	.164								
Communal Orientation	.24	.19	[.05, .43]	.012								
Communion	.24	.21	[.08, .39]	.003								
Unmitigated Communion	-.14	-.14	[-.27, -.01]	.037								
Empathic Concern	.13	.13	[-.02, .27]	.009								
Gender	-.03	-.02	[-.16, .10]	.645								
Compassionate Goals	.38	.34	[.24, .53]	< .001								
DV: Responsiveness												
Covariates Entered in Separate Analyses												
Compassionate Love	.23	.28	[.14, .33]	< .001	.06	.05	[-.05, .18]	.272	.43	.40	[.30, .55]	< .001
Communal Orientation	.46	.38	[.33, .59]	< .001	.01	.01	[-.10, .12]	.848	.40	.37	[.29, .51]	< .001
Communion	.46	.44	[.36, .57]	< .001	.01	.01	[-.10, .12]	.845	.39	.36	[.28, .49]	< .001
Unmitigated Communion	.13	.14	[.03, .23]	.013	.09	.07	[-.03, .20]	.141	.53	.50	[.41, .64]	< .001
Empathic Concern	.31	.35	[.22, .41]	< .001	.01	.00	[-.11, .12]	.929	.43	.41	[.32, .54]	< .001
Covariates Entered in a Single Analysis												
Compassionate Love	.03	.04	[-.08, .14]	.569								
Communal Orientation	.21	.17	[.06, .36]	.006								
Communion	.35	.33	[.22, .47]	< .001								
Unmitigated Communion	-.09	-.09	[-.19, .01]	.088								
Empathic Concern	.10	.11	[-.02, .21]	.1065								
Gender	-.04	-.03	[-.14, .07]	.470								

Compassionate Goals	.32	.30	[.21, .43]	< .001

N = 318 for all analyses

Supplemental Table 5.

Study 3 Analyses Predicting Other-Focused Reasons for Giving and Gratitude, Controlling for Gender

	b	95%CI	*pr*	*p*
DV: Other-Focused Reasons for Giving				
Covariates Entered in Separate Analyses				
Self-Focused Reasons for Giving	.16	[.07, .24]	.25	< .001
Compassionate Love	.07	[-.07, .20]	.07	.337
Gender	.33	[.12, .54]	.31	.002
Compassionate Goals	.38	[.18, .57]	.27	< .001
Self-Focused Reasons for Giving	.15	[.07, .24]	.24	.001
Communal Orientation	-.03	[-.20, .14]	-.03	.706
Gender	.32	[.11, .54]	.30	.004
Compassionate Goals	.44	[.28, .61]	.36	< .001
Self-Focused Reasons for Giving	.16	[.07, .24]	.14	< .001
Communion	-.03	[-.20, .15]	-.04	.776
Gender	.32	[.11, .54]	.18	.003
Compassionate Goals	.45	[.27, .62]	.34	< .001
Self-Focused Reasons for Giving	.16	[.07, .24]	.25	< .001
Unmitigated Communion	.08	[-.06, .23]	.08	.259
Gender	.32	[.12, .53]	.30	.003
Compassionate Goals	.39	[.22, .56]	.31	< .001
Self-Focused Reasons for Giving	.17	[.08, .25]	.27	< .001
Empathic Concern	.12	[-.04, .28]	.11	.130
Gender	.31	[.10, .51]	.29	.004
Compassionate Goals	.37	[.20, .55]	.29	< .001
Covariates Entered in a Single Analysis				
Self-Focused Reasons for Giving	.15	[.06, .24]	.24	.001
Compassionate Love	.03	[-.13, .18]	.03	.709
Communal Orientation	-.09	[-.28, .10]	-.07	.347
Communion	-.13	[-.34, .09]	-.09	.244
Unmitigated Communion	.09	[-.08, .26]	.08	.312
Empathic Concern	.16	[-.04, .36]	.11	.115
Gender	.32	[.10, .54]	.30	.004
Compassionate Goals	.36	[.16, .57]	.25	.001
DV: Gratitude				
Covariates Entered in Separate Analyses				
Compassionate Love	.05	[-.07, .17]	.06	.438
Gender	.33	[.13, .52]	.32	.001
Compassionate Goals	.38	[.21, .55]	.30	< .001
Communal Orientation	.02	[-.13, .16]	.02	.823
Gender	.29	[.09, .48]	.28	.004
Compassionate Goals	.41	[.27, .55]	.39	< .001

Communion	.22	[.07, .36]	.22	.004
Gender	.31	[.12, .50]	.31	.002
Compassionate Goals	.31	[.16, .46]	.28	< .001
Unmitigated Communion	-.04	[-.17, .09]	-.05	.530
Gender	.32	[.13, .51]	.32	.001
Compassionate Goals	.44	[.29, .59]	.39	< .001
Empathic Concern	.13	[-.01, .26]	.13	.065
Gender	.30	[.12, .49]	.31	.002
Compassionate Goals	.35	[.20, .50]	.31	< .001
Covariates Entered in a Single Analysis				
Compassionate Love	.02	[-.11, .15]	.03	.734
Communal Orientation	-.09	[-.25, .07]	.09	.248
Communion	.24	[.07, .42]	.22	.007
Unmitigated Communion	-.10	[-.24, .04]	-.11	.149
Empathic Concern	.09	[-.08, .25]	.07	.320
Gender	.28	[.09, .47]	.28	.004
Compassionate Goals	.31	[.14, .48]	.25	.001

Supplement to Section 4.2.4 Compassionate Goals, Other-Focused Reasons for Giving, and Friend's Gratitude.

We tested the path model in which participants' compassionate goals indirectly predicted friends' gratitude through participants' other-focused reasons for giving, controlling for the other measures of prosocial orientations. When we regressed participants' other-focused reasons for giving on their compassionate goals, controlling for their self-focused reasons for giving, compassionate love, communal orientation, communion, unmitigated communion, and empathic concern, participants' compassionate goals predicted their greater other-focused reasons for giving, $b = .38$, $SE = .11$, $t(188.82) = 3.55$, 95% CI [.17, .58], $p < .001$, $pr = .25$. Full results from this analysis appears in the bottom right section of Table 7 in the manuscript. When we regressed friends' gratitude on participants' other-focused reasons for giving and compassionate goals controlling for their self-focused reasons for giving, compassionate love, communal orientation, communion, unmitigated communion, and empathic concern, participants' friend-centered reasons for giving predicted friends' gratitude, $b = .28$, $SE = .07$, $t(182.54) = 4.15$, 95% CI [.15, .41], $p < .001$, $pr = .29$. (Full results from this analysis appear in Table 3 below). The 95% confidence interval for the indirect effect did not include zero, 95% CI [.04, .20], again, indicating a significant indirect effect of compassionate goals on friends' gratitude through participants' other-focused reasons for giving, when we controlled for other measures of prosocial orientation.

Table 6

Study 3 Supplemental Analyses Examining Participants' Other-Focused Reasons for Giving, Controlling for Participants' Compassionate Goals, Self-Focused Reasons for Giving, and Other Measures of Prosocial Orientations.

	Outcome: Friends' Gratitude			
	b	95%CI	*pr*	*p*
Participants':				
Self-Focused Reasons for Giving	-.00	[-.09, .08]	-.01	>.250
Compassionate Love	-.13	[-.27, .02]	-.13	.081
Communal Orientation	.13	[-.05, .32]	.11	.144
Communion	-.06	[-.27, .15]	-.05	>.250
Unmitigated Communion	-.04	[-.21, .12]	-.04	>.250
Empathic Concern	.19	[-.00, .38]	.15	.050
Compassionate Goals	.04	[-.16, .24]	.03	>.250
Other-Focused Reasons for Giving	.28	[.15, .41]	.29	<.001

ALL OF THE FOLLOWING MEASURES WERE INCLUDED IN ALL STUDIES

Compassionate and Self-Image Goals

In general, how much do you *want* or *try* to:		Not at All	A little	Somewhat	A lot	Extremely
1.	avoid appearing ignorant, incompetent, or unintelligent	1	2	3	4	5
2.	be constructive in your comments to others	1	2	3	4	5
3.	get others to notice your positive qualities	1	2	3	4	5
4.	avoid doing anything that would be harmful to others	1	2	3	4	5
5.	give the appearance of being on top of things.	1	2	3	4	5
6.	appear helpful.	1	2	3	4	5
7.	avoid being criticized by others	1	2	3	4	5
8.	avoid being selfish or self-centered	1	2	3	4	5
9.	have compassion for others' mistakes and weaknesses	1	2	3	4	5
10.	avoid appearing unlikable	1	2	3	4	5
11.	understand how your actions affect others	1	2	3	4	5
12.	avoid focusing on your own needs to the exclusion of others' needs	1	2	3	4	5
13.	get others to recognize or acknowledge your intelligence	1	2	3	4	5
14.	be supportive of others	1	2	3	4	5
15.	avoid doing things that aren't helpful to you or others	1	2	3	4	5
16.	convince others that you are right	1	2	3	4	5
17.	make a positive difference in someone else's life	1	2	3	4	5
18.	avoid taking risks or making mistakes	1	2	3	4	5
19.	do things that are helpful for both you and others.	1	2	3	4	5
20.	avoid being wrong	1	2	3	4	5
21.	do things you knew you could succeed at	1	2	3	4	5
22.	avoid neglecting your relationship with others	1	2	3	4	5
23.	avoid showing your weaknesses	1	2	3	4	5
24.	avoid shutting people off emotionally	1	2	3	4	5

Compassionate Love

Please rate the following items:		Not at All True of Me				Very True of Me
1.	When I see people I do not know feeling sad, I feel a need to reach out to them.	1	2	3	4	5
2.	I spend a lot of time concerned about the well-being of humankind.	1	2	3	4	5

3.	When I hear about someone (a stranger) going through a difficult time, I feel a great deal of compassion for him or her.	1	2	3	4	5
4.	It is easy for me to feel the pain (and joy) experienced by others, even though I do not know them.	1	2	3	4	5
5.	If I encounter a stranger who needs help, I would do almost anything I could to help him or her.	1	2	3	4	5
6.	I feel considerable compassionate love for people from everywhere.	1	2	3	4	5
7.	I would rather suffer myself than see someone else (a stranger) suffer.	1	2	3	4	5
8.	If given the opportunity, I am willing to sacrifice in order to let people from other places who are less fortunate achieve their goals.	1	2	3	4	5
9.	I tend to feel compassion for people, even though I do not know them.	1	2	3	4	5
10.	One of the activities that provides me with the most meaning in my life is helping others in the world when they need help.	1	2	3	4	5
11.	I would rather engage in actions that help others, even though they are strangers, than engage in actions that would help me.	1	2	3	4	5
12.	I often have tender feelings toward people (strangers) when they seem to be in need.	1	2	3	4	5
13.	I feel a selfless caring for most of humankind.	1	2	3	4	5
14.	I accept others whom I do not know even when they do things I think are wrong.	1	2	3	4	5
15.	If a person (a stranger) is troubled, I usually feel extreme tenderness and caring.	1	2	3	4	5
16.	I try to understand rather than judge people who are strangers to me.	1	2	3	4	5
17.	I try to put myself in a stranger’s shoes when he or she is in trouble.	1	2	3	4	5
18.	I feel happy when I see that others (strangers) are happy.	1	2	3	4	5
19.	Those whom I encounter through my work and public life can assume that I will be there if they need me.	1	2	3	4	5
20.	I want to spend time with people I don’t know well so that I can find ways to help enrich their lives.	1	2	3	4	5
21.	I very much wish to be kind and good to fellow human beings.	1	2	3	4	5

Communal Orientation Scale

Please rate how characteristic each item is of you, using the scale provided.		Extremely Uncharacteristic				Extremely Characteristic
1.	It bothers me when other people neglect my needs.	1	2	3	4	5
2.	When making a decision, I take other people's needs and feelings into account.	1	2	3	4	5
3.	I'm not especially sensitive to other people's feelings.	1	2	3	4	5
4.	I don't consider myself to be a particularly helpful person.	1	2	3	4	5
5.	I believe people should go out of their way to be helpful.	1	2	3	4	5
6.	I don't especially enjoy giving others aid.	1	2	3	4	5
7.	I expect people I know to be responsive to my needs and feelings.	1	2	3	4	5
8.	I often go out of my way to help another person.	1	2	3	4	5
9.	I believe it's best not to get involved taking care of other people's personal needs.	1	2	3	4	5
10.	I'm not the sort of person who often comes to the aid of others.	1	2	3	4	5
11.	When I have a need, I turn to others I know for help.	1	2	3	4	5
12.	When people get emotionally upset, I tend to avoid them.	1	2	3	4	5
13.	People should keep their troubles to themselves.	1	2	3	4	5
14.	When I have a need that others' ignore, I'm hurt.	1	2	3	4	5

AGENCY AND COMMUNION

INSTRUCTIONS: The items below consist of a pair of contradictory characteristics--that is, you cannot be both at the same time. The numbers form a scale between the two extremes. You are to circle the number that describes where you fall on the scale.

Not at all arrogant	1	2	3	4	5	Very arrogant
Not at all independent	1	2	3	4	5	Very independent
Not at all emotional	1	2	3	4	5	Very emotional
Looks out for self	1	2	3	4	5	Looks out for others
Very passive	1	2	3	4	5	Very active
Not at all egotistical	1	2	3	4	5	Very egotistical
Difficult to devote self completely to others	1	2	3	4	5	Easy to devote self completely to others
Very rough	1	2	3	4	5	Very gentle

Not at all helpful to others	1	2	3	4	5	Very helpful to others
Not at all boastful	1	2	3	4	5	Very boastful
Not at all competitive	1	2	3	4	5	Very competitive
Not at all kind	1	2	3	4	5	Very kind
Not at all aware of others' feelings	1	2	3	4	5	Very aware of others' feelings
Can make decisions easily	1	2	3	4	5	Has difficulty making decisions
Not at all greedy	1	2	3	4	5	Very greedy
Gives up easily	1	2	3	4	5	Never gives up
Not at all self-confident	1	2	3	4	5	Very self-confident
Feels very inferior	1	2	3	4	5	Feels very superior
Not at all dictatorial	1	2	3	4	5	Very dictatorial
Not at all understanding of others	1	2	3	4	5	Very understanding of others
Not at all cynical	1	2	3	4	5	Very cynical
Very cold in relations with others	1	2	3	4	5	Very warm in relations with others
Not at all hostile	1	2	3	4	5	Very hostile
Goes to pieces under pressure	1	2	3	4	5	Stands up well under pressure

Unmitigated Communion Scale

INSTRUCTIONS: Read each item carefully. Using the scale, choose the number that best describes you.

		**Strongly Disagree**	**Slightly Disagree**	**Neither Agree nor Disagree**	**Slightly Agree**	**Strongly Agree**
1.	I *always* place the needs of others above my own.	1	2	3	4	5
2.	I never find myself getting overly involved in others' problems.	1	2	3	4	5
3.	For me to be happy, I need others to be happy.	1	2	3	4	5
4.	I worry about how other people get along without me when I am not there.	1	2	3	4	5
5.	I have *no* trouble getting to sleep at night when other people are upset.	1	2	3	4	5
6.	It is impossible for me to satisfy my own needs when they interfere with the needs of others.	1	2	3	4	5
7.	I can't say no when someone asks for help.	1	2	3	4	5
8.	Even when I am exhausted, I will always help other people.	1	2	3	4	5
9.	I often worry about others' problems.	1	2	3	4	5

Empathic Concern

INSTRUCTIONS: The following statements inquire about your thoughts and feelings in a variety of situations. For each item, indicate how well it describes you by choosing the appropriate number. READ EACH ITEM CAREFULLY BEFORE RESPONDING. Answer as honestly as you can.

		**Does not describe me well**				**Describes me very well**
1.	I often have tender, concerned feelings for people less fortunate than me.	1	2	3	4	5
2.	Sometimes I don't feel very sorry for other people when they are having problems.	1	2	3	4	5
3.	When I see someone being taken advantage of, I feel kind of protective towards them.	1	2	3	4	5
4.	Other people's misfortunes do not usually disturb me a great deal.	1	2	3	4	5
5.	When I see someone being treated unfairly, I sometimes don't feel very much pity for them.	1	2	3	4	5
6.	I am often quite touched by things that I see happen.	1	2	3	4	5
7.	I would describe myself as a pretty soft-hearted person.	1	2	3	4	5

Social Desirability (Included in Studies 1 and 2 only)

**INSTRUCTIONS**: Listed below are a number of statements concerning personal attitudes and traits. Read each item and decide whether the statement is true (T) or false (F) as it pertains to you personally. It's best to go with your first judgment and not spend too long mulling over any one question.		**True**	**False**
1.	Before voting, I thoroughly investigate the qualifications of the candidates		
2.	I never hesitate to go out of my way to help someone in trouble.		
3.	It is sometimes hard for me to go on with my work if I am not encouraged.		
4.	I have never intensely disliked anyone.		
5.	On occasions I have had doubts about my ability to succeed in life.		
6.	I sometimes feel resentful when I don’t get my way.		
7.	I am always careful about my manner of dress.		
8.	My table manners at home are as good as when I eat out in a restaurant.		
9.	If I could get into a movie without paying and be sure I was not seen I would probably do it.		
10.	On a few occasions, I have given something up because I thought too little of my ability.		
11.	I like to gossip at times.		

12.	There have been times when I felt like rebelling against people in authority even though I knew they were right.		
13.	No matter who I'm talking to, I'm always a good listener.		
14.	I can remember "playing sick" to get out of something.		
15.	There have been occasions when I have taken advantage of someone.		
16.	I'm always willing to admit it when I make a mistake.		
17.	I always try to practice what I preach.		
18.	I don't find it particularly difficulty to get along with loudmouthed, obnoxious people.		
19.	I sometimes try to get even rather than forgive and forget.		
20.	When I don't know something I don't mind at all admitting it.		
21.	I am always courteous, even to people who are disagreeable.		
22.	At times I have really insisted on having things my own way.		
23.	There have been occasions when I felt like smashing things.		
24.	I would never think of letting someone else be punished for my wrong-doings.		
25.	I never resent being asked to return a favor.		
26.	I have never been irked when people express ideas very different from my own.		
27.	I never make a long trip without checking the safety of my car.		
28.	There have been times when I was quite jealous of the good fortune of others.		
29.	I have almost never felt the urge to tell someone off.		
30.	I am sometimes irritated by people who ask favors of me.		
31.	I have never felt that I was punished without cause.		
32.	I sometimes think when people have a misfortune they only got what they deserved.		
33.	I have never deliberately said something that hurt someone's feelings.		

BACKGROUND INFORMATION

The following items ask about you and your context to help us interpret the results of the survey.

1. Your Gender: _____Male _____Female
2. Your Race (check all that apply):
   _____ American Indian/Alaska Native _____Black or African American
   _____Asian _____Caucasian/White
   _____Native Hawaiian or other pacific islander ________Other
3. Your ethnicity
   _____Hispanic/Latino (any race)
   _____Not Hispanic/Latino
4. Your Age: ______

INCLUDED IN STUDY 1 ONLY

Altruism Scale

	Please rate how often you do each of the following.	Never				Very Often
1.	I have helped push a stranger's car out of the snow.	1	2	3	4	5
2.	I have given directions to a stranger.	1	2	3	4	5
3.	I have made change for a stranger.	1	2	3	4	5
4.	I have given money to a charity.	1	2	3	4	5
5.	I have given money to a stranger who needed it (or asked me for it).	1	2	3	4	5
6.	I have donated goods or clothes to a charity.	1	2	3	4	5
7.	I have done volunteer work for a charity.	1	2	3	4	5
8.	I have donated blood.	1	2	3	4	5
9.	I have helped carry a stranger's belongings (books, parcels, etc.)	1	2	3	4	5
10.	I have delayed an elevator and held the door open for a stranger.	1	2	3	4	5
11.	I have allowed someone to go ahead of me in a line (at a copy machine, in the supermarket, etc).	1	2	3	4	5
12.	I have given a stranger a lift in my car.	1	2	3	4	5
13.	I have pointed out a clerk's error (in a bank, at the supermarket) in undercharging me for an item.	1	2	3	4	5
14.	I have let a neighbor whom I didn't know too well borrow an item of some value to me (e.g., a dish, tools, etc).	1	2	3	4	5
15.	I have bought "charity" Christmas cards deliberately because I knew it as a good cause.	1	2	3	4	5
16.	I have helped a classmate who I did not know that well with a homework assignment when my knowledge was greater than his or hers.	1	2	3	4	5
17.	I have before being asked, voluntarily looked after a neighbor's pets or children without being paid for it.	1	2	3	4	5
18.	I have offered to help a handicapped or elderly stranger across a street.	1	2	3	4	5
19.	I have offered my seat on a bus or train to a stranger who was standing.	1	2	3	4	5
20.	I have helped an acquaintance to move households.	1	2	3	4	5

INCLUDED IN STUDY 2 ONLY

Social Support Made Available to Others

		Very Strongly Disagree				Very Strongly Agree
1.	I am around for a special person in my life when he/she is in need	1	2	3	4	5
2.	I am someone with whom a special person in my life can share his/her joys and sorrows	1	2	3	4	5
3.	I really try to help my family	1	2	3	4	5
4.	I provide emotional help and support to my family members	1	2	3	4	5
5.	I am a real source of comfort for the special person in my life	1	2	3	4	5
6.	I really try to help my friends	1	2	3	4	5
7.	My friends can count on me when things go wrong	1	2	3	4	5
8.	My family members can talk about their problems with me	1	2	3	4	5
9.	My friends feel that I am someone with whom they can share their joys and sorrows	1	2	3	4	5
10.	There is a special person in my life who believes that I care about his/her feelings	1	2	3	4	5
11.	I am willing to help my family members make decisions	1	2	3	4	5
12.	My friends can talk about their problems with me	1	2	3	4	5

Responsiveness to Others

		Not at all				Very much
1.	I do things to show others that I care about them	1	2	3	4	5
2.	I behave warmly toward others	1	2	3	4	5
3.	I really listen to others when they speak	1	2	3	4	5
4.	I see things from others' points of view	1	2	3	4	5
5.	I make others feel comfortable about themselves and how they feel	1	2	3	4	5
6.	I am sensitive to others ' feelings	1	2	3	4	5
7.	I am uncaring toward others	1	2	3	4	5
8.	I show respect for others' capabilities and talents	1	2	3	4	5
9.	I don't really take others' concerns seriously	1	2	3	4	5
10.	I am sincere when I interact with others	1	2	3	4	5
11.	I make others feel valued as people	1	2	3	4	5
12.	I understand others' concerns	1	2	3	4	5

INCLUDED IN STUDY 3 ONLY

Reasons for Giving

Describe how you try to be helpful, generous, thoughtful, or supportive of ___. Include what you do, why you do it, and how often you do those things?

Rate the importance of each of the following reasons for your decision to do things for ___. (please write your friend's initials in the blank space above):		Not at All Important				Extremely Important
1.	Because I care about ___.	1	2	3	4	5
2.	To avoid contributing to my friend's emotional distress.	1	2	3	4	5
3.	Because I thought it would make me feel better.	1	2	3	4	5
4.	To avoid feeling guilty.	1	2	3	4	5
5.	Because I thought it would be helpful or supportive for ____.	1	2	3	4	5
6.	To avoid neglecting my friend's needs.	1	2	3	4	5
7.	To avoid conflict in my friendship.	1	2	3	4	5
8.	So ___ would appreciate or like me.	1	2	3	4	5
9.	To improve ____'s life a little.	1	2	3	4	5
10.	Because I did not want my friend to be hurt.	1	2	3	4	5
11.	To prove to ___ that I am a good friend.	1	2	3	4	5
12.	To prevent my friend from becoming upset.	1	2	3	4	5
13.	Because ___ needed a friend.	1	2	3	4	5
14.	To prevent my friend from losing interest in me as a friend.	1	2	3	4	5
15.	To show ____ that I am a good person.	1	2	3	4	5
16.	To prevent my friend from getting angry at me.	1	2	3	4	5

Gratitude toward Partner

Describe how _____ is helpful, generous, thoughtful, or supportive of you. Include what does he/she do, how does it make you feel, and how often does ___ do those things?

**People often feel different things in different situations. When ___ does things for you, how much do you feel?** (please write your friend's initials in the blank space above)**:**		**Not at All**				**Extremely**
1.	Thankful	1	2	3	4	5
2.	Happy	1	2	3	4	5
3.	Indebted	1	2	3	4	5
4.	Proud	1	2	3	4	5
5.	Resentful	1	2	3	4	5
6.	Grateful	1	2	3	4	5
7.	Admiring	1	2	3	4	5
8.	Anger	1	2	3	4	5
9.	Sadness	1	2	3	4	5
10.	Appreciative	1	2	3	4	5
11.	Frustrated	1	2	3	4	5
12.	Peaceful	1	2	3	4	5
13.	Clear	1	2	3	4	5
14.	Close	1	2	3	4	5
15.	Anxious	1	2	3	4	5
16.	Conflicted	1	2	3	4	5
17.	Confused	1	2	3	4	5
